# Supplementary material for: The interactome of intact mitochondria by cross-linking mass spectrometry provides evidence for coexisting respiratory supercomplexes
Source: Mol Cell Proteomics. 2017 Dec 8;17(2):216–32. doi: 10.1074/mcp.RA117.000470 (PMC5795388; doi:10.1074/mcp.RA117.000470)
Supplement: Supplemental Data [file supp_RA117.000470_133922_0_supp_23978_fzffwf.zip › spectra_annotation/mito_spectra_annotation/499-1-6-1-9-1.pdf]

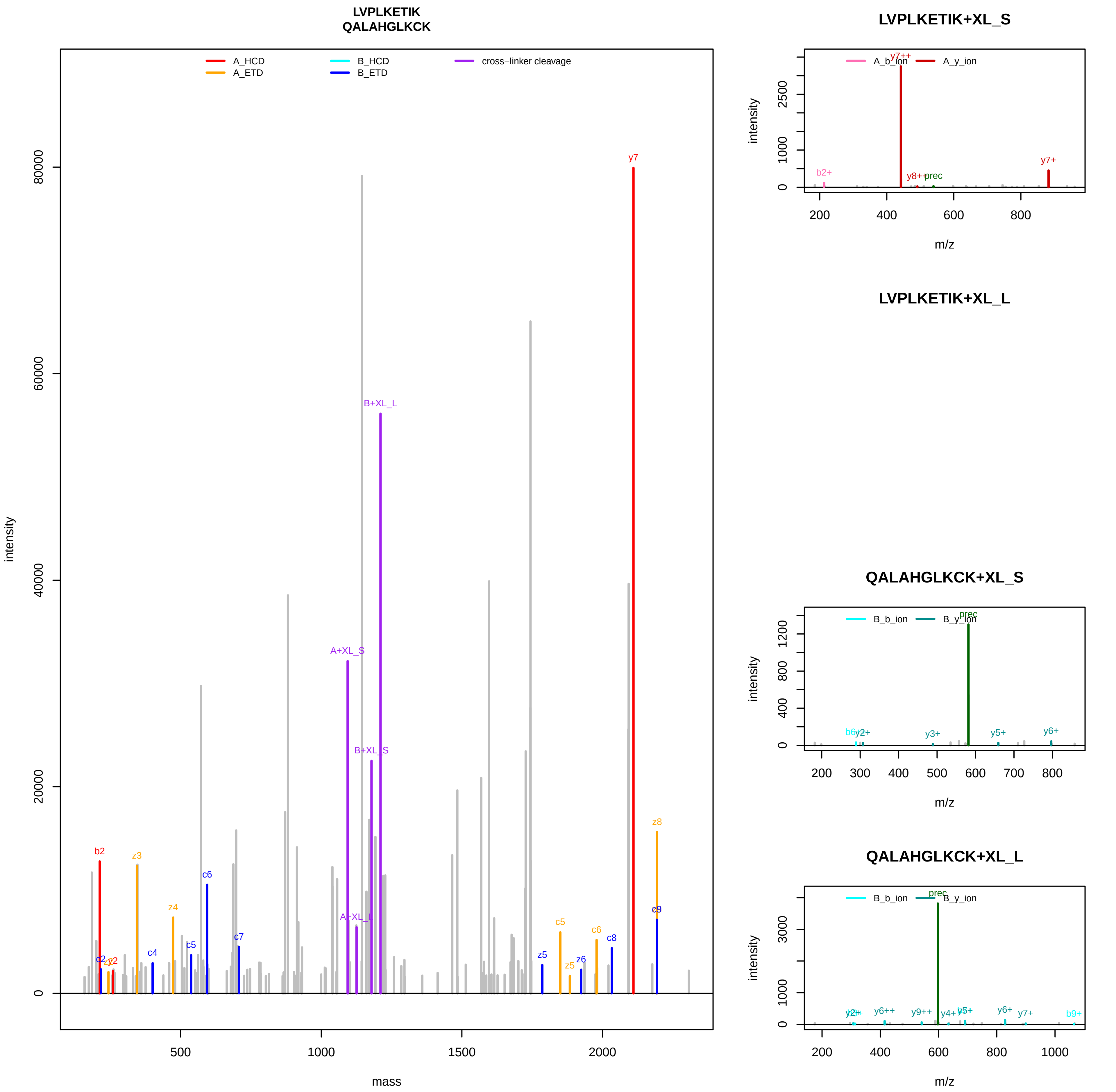

LVPLKETIK
QALAHGLKCK
A_HCD
A_ETD
B_HCD
B_ETD
cross−linker cleavage
intensity
mass
0
20000
40000
60000
80000
500
1000
1500
2000
b2
c2
z2
y2
z3
c4
z4
c5
c6
c7
A+XL_S
A+XL_L
B+XL_S
B+XL_L
z5
c5
z5
z6
c6
c8
y7
z8
c9
LVPLKETIK+XL_S
A_b_ion
A_y_ion
y7++
b2+
y8++
prec
y7+
m/z
LVPLKETIK+XL_L
QALAHGLKCK+XL_S
B_b_ion
B_y_ion
prec
b6
y2+
y3+
y5+
y6+
QALAHGLKCK+XL_L
y2+
y6++
y9++
y4+
y5+
y6+
y7+
b9+
